# Local osteo-enhancement of osteoporotic vertebra with a triphasic bone implant material increases strength—a biomechanical study

**DOI:** 10.1007/s00402-020-03382-x

**Published:** 2020-02-27

**Authors:** Matthias Trost, Werner Schmoelz, Doris Wimmer, Romed Hörmann, Sönke Frey, Tobias Ludger Schulte

**Affiliations:** 1 Department of Orthopaedics and Traumatology, St. Josef-Hospital, Ruhr University Bochum, Bochum, Germany; 2 Department of Trauma Surgery, Medical University of Innsbruck, Innsbruck, Austria; 3 Division of Clinical and Functional Anatomy, Department of Anatomy, Medical University of Innsbruck, Innsbruck, Austria

**Keywords:** Osteoporosis, Spine, Vertebral augmentation, Triphasic bone implant material, Biomechanical study

## Abstract

**Purpose:**

The aim of this study was to assess the biomechanical properties of intact vertebra augmented using a local osteo-enhancement procedure to inject a triphasic calcium sulfate/calcium phosphate implant material.

**Methods:**

Twenty-one fresh frozen human cadaver vertebra (Th11–L2) were randomized into three groups: treatment, sham, and control (*n* = 7 each). Treatment included vertebral body access, saline lavage to displace soft tissue and marrow elements, and injection of the implant material to fill approximately 20% of the vertebral body by volume. The sham group included all treatment steps, but without injection of the implant material. The control group consisted of untreated intact osteoporotic vertebra. Load at failure and displacement at failure for each of the three groups were measured in axial compression loading.

**Results:**

The mean failure load of treated vertebra (4118 N) was significantly higher than either control (2841 N) or sham (2186 N) vertebra (*p* < 0.05 for: treatment vs. control, treatment vs. sham). Treated vertebra (1.11 mm) showed a significantly higher mean displacement at failure than sham vertebra (0.80 mm) (*p* < 0.05 for: treatment vs. sham). In the control group, the mean displacement at failure was 0.99 mm.

**Conclusions:**

This biomechanical study shows that a local osteo-enhancement procedure using a triphasic implant material significantly increases the load at failure and displacement at failure in cadaveric osteoporotic vertebra.

## Introduction

Osteoporotic vertebral compression fractures directly affect the quality of life for patients and are becoming an increasing burden to national healthcare systems with an aging population [1]. Fractures are treated conservatively with analgesics, bracing and physiotherapy, or surgically with vertebroplasty, kyphoplasty, or elastoplasty using non-resorbable polymeric implant materials [2]. While these treatments address the index fracture, a patient’s fracture risk remains high, particularly if they have more than one prevalent vertebral fracture or if the vertebral fracture is severe [3, 4]. Current osteoporotic fracture prevention strategies include public health programs to improve daily behavior, such as regular exercise, smoking cessation, and diet, as well as the use of systemic pharmaceuticals such as bisphosphonates and parathyroid hormone [5–7]. There is a growing consensus that new and innovative osteoporotic fracture prevention strategies are required to effectively manage the rise in healthcare costs associated with osteoporotic fractures.

A recent review of osteoporotic therapies highlighted the potential benefits of using a new minimally invasive approach to address osteoporotic bone loss and reduce fracture risk [8]. A local osteo-enhancement procedure utilizing resorbable, triphasic calcium sulfate/calcium phosphate injectable implant material is under clinical investigation as a means of preventing osteoporotic hip fractures. The implant material increased the fracture strength of cadaveric osteoporotic femurs [9]. This approach to strengthening at risk osteoporotic vertebra has not been previously studied.

The aim of this study was to determine whether local osteo-enhancement using a triphasic bone implant material improves the initial biomechanical properties of osteoporotic vertebra. This pilot study used fresh frozen cadaveric human osteoporotic vertebra to compare the immediate effects of the treatment procedure to a sham procedure and to untreated control vertebra on load at failure and stiffness.

## Materials and methods

### Specimen preparation

Twenty-one fresh frozen human cadaver vertebra (Th11–L2) from consenting informed donors were included in the study (Table 1). The bone mineral density (BMD) of each vertebra was measured using a qCT scan (LightSpeed VCT 16, GE Healthcare, Chicago, USA) with a calibration phantom (European Forearm Phantom, QRM GmbH, Möhrendorf, Germany) [10]. For BMD measurement, the average of three circular regions of interest placed in the trabecular area of three slices evenly distributed across the height of each vertebral body was taken. Previously fractured vertebra were excluded.

**Table 1:** Study population

	Control	Sham	Treatment
*N* (total)	7	7	7
*N* (male)	3	3	3
*N* (female)	4	4	4
*N* (Th11)	1	1	1
*N* (Th12)	3	4	4
*N* (L1)	3	1	2
*N* (L2)	0	1	0
Age (years)	74 (64–85)	75 (63–94)	81 (69–94)

Mean and range are stated for the age of the specimens

All soft tissues were removed from individual vertebra and the volume of each vertebra was measured using water displacement at room temperature. Vertebral endplates were potted using polymethylmethacrylate (PMMA) (Technovit^®^ 3040, Heraeus Kulzer GmbH, Wehrheim, Germany) to ensure a uniform stress concentration across the endplates during axial loading [11, 12]. The caudal portion of the facet joint was resected to avoid load transfer through the posterior vertebral structures. Vertebra were stored at –20 °C. Prior to experiments, vertebra were thawed at 6 °C for 20 h and equilibrated to room temperature for 4 h.

### Initial stiffness testing

**Fig. 1:**
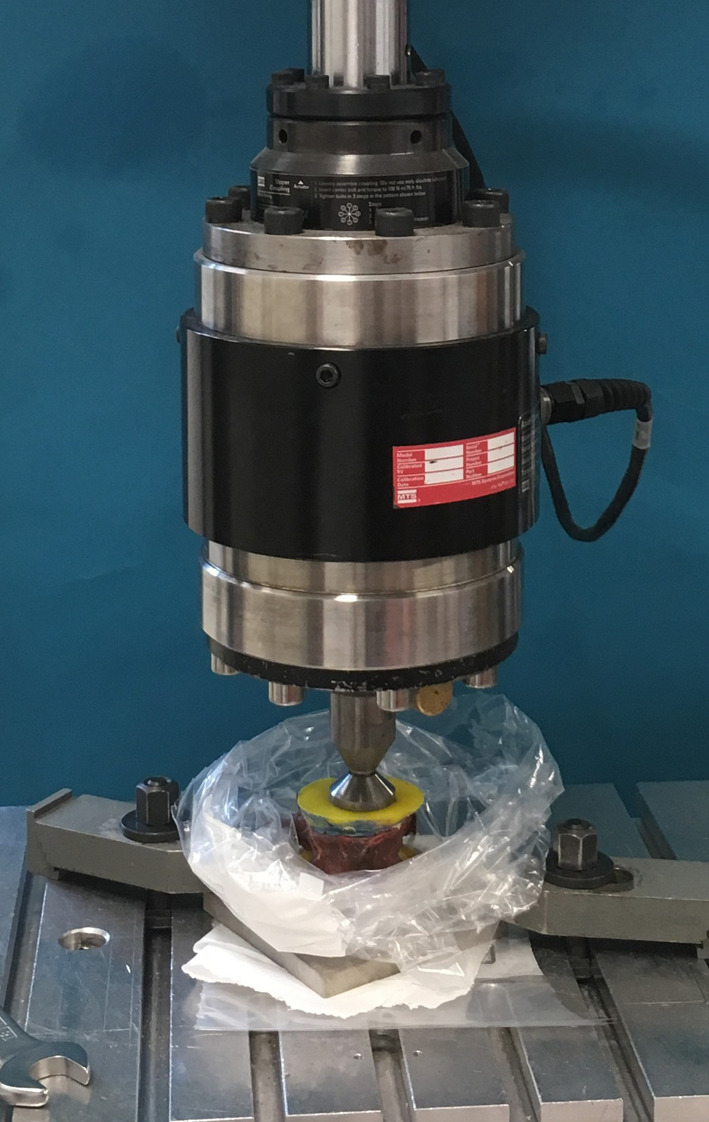
The experimental set-up for mechanical testing in the materials testing machine

Before any intervention, the elastic stiffness of all vertebra was measured in axial loading at room temperature with a servo hydraulic materials testing machine (858 Mini Bionix II, MTS, Eden Prairie, Minnesota, USA). The load was induced across the central area of the cranial endplate with a ball and socket joint [13]. Specimens were preconditioned from 20 to 200 N for 20 cycles (0.5 Hz) and subsequently subjected to a non-destructive load ranging from 100 to 500 N (ramp 0.1 mm/s, for three cycles) to determine the elastic stiffness (Fig. 1) [14].

### Treatment procedure

**Fig. 2:**
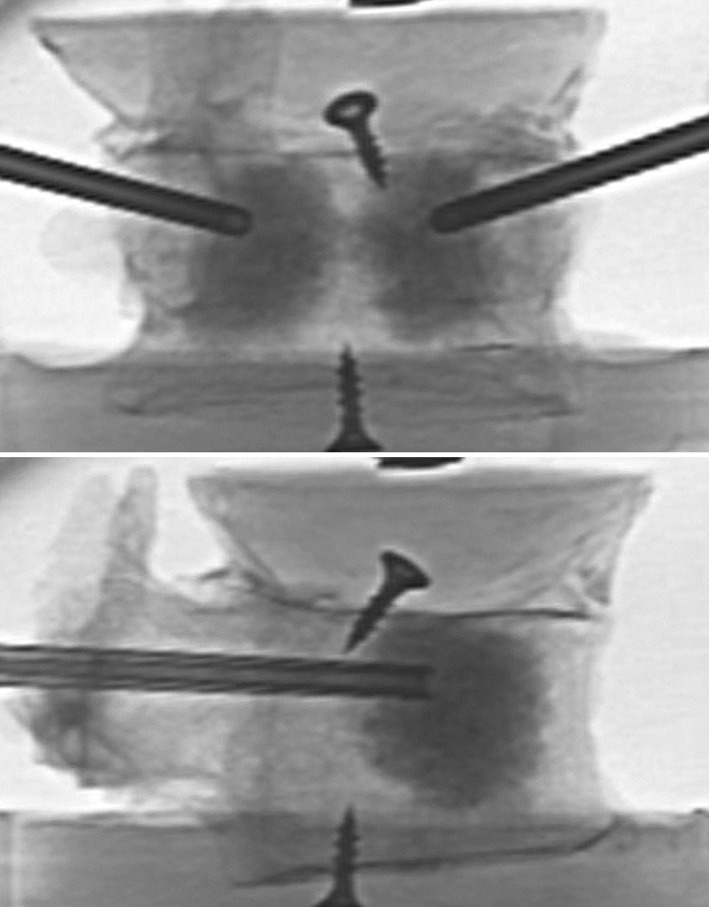
Radiographs in anteroposterior and lateral projections after augmentation of the vertebral body with the triphasic bone implant material AGN1

Following the measurement of initial elastic stiffness, the randomly assigned specimens were segregated into one of three groups (*n* = 7 for each group): treatment, sham, and control. Treatment included access, preparation of the site using saline lavage, and injection of the implant material. Two 11G bone access cannulas were placed in the anterior half of the vertebral body through the pedicles. Saline lavage was performed as described by Hoppe et al. by first attaching one 20 mL syringe containing 10 mL of saline to one access cannula while an empty 20 mL syringe was attached to the contralateral cannula [15]. A slight vacuum was then applied to the empty syringe as gentle pressure was applied to inject saline from the opposite syringe to pull marrow, fat, and soft tissue from the vertebral body. The process was repeated for a total of four times using a total of 40 mL of saline. Triphasic implant material consisting of 75% calcium sulfate and 25% biphasic calcium phosphates (AGN1, AgNovos Healthcare, Rockville, USA) was then mixed and manually injected under fluoroscopic guidance through 14G delivery cannulas placed coaxial to the 11G access cannulas. The implant material was delivered through both pedicles to distribute the implant material equally in the anterior half of the vertebral body. The injection procedure was stopped upon reaching the target fill of 20% by volume [11, 13, 14, 16]. After treatment, radiographs were taken in anteroposterior and lateral projections to verify correct implant positioning (Fig. 2). The sham group included all steps described in the treatment group; however, no implant material was injected. The control group consisted of intact osteoporotic vertebra with no further experimental manipulation. After surgical manipulation, all vertebrae were kept at room temperature for several hours to ensure that the implant material was completely set before transfer to 6 °C. Vertebrae were stored for 20 h prior to performing the final biomechanical testing with loading to failure to measure the ultimate strength of each specimen.

### Secondary stiffness and load to failure testing

Before mechanical testing of the three groups, the vertebra were equilibrated to room temperature for 4 h. A second measurement of the elastic stiffness was performed using the same parameters as the initial stiffness testing. Following stiffness measurements, specimens were loaded to failure with a displacement rate of 0.5 mm/s. Force and displacement data were recorded at 100 Hz [17].

### Statistical analysis

Statistical analysis was carried out using IBM SPSS Statistics, version 24.0 (IBM Corporation, Armonk, New York, USA). The initial and second elastic stiffness of each vertebra was compared using a paired *t*-test. Failure load was defined as the first negative slope of the force displacement curve between two measurement points. The three groups were compared using a one-way ANOVA followed by a Tukey post-hoc test. Statistical significance was set at the 5% level (*p* ≤ 0.05).

## Results

Prior to treatment, the mean bone mineral density did not differ significantly between the three groups: control was 62.5 mg/cm^3^ (48.6–77.6 mg/cm^3^), sham was 57.6 mg/cm^3^ (43.1–77.9 mg/cm^3^), and treatment was 56.5 mg/cm^3^ (44.9–70.8 mg/cm^3^) (*p* = 0.55) (Table 1). The mean vertebral body volumes also did not differ significantly between the groups: control was 29.5 mL (18.9–40.3 mL), sham was 31.8 mL (21.1–38.8 mL), and treatment was 34.4 mL (28.4–44.5 mL) (*p* = 0.39) (Table 1).

The initial elastic stiffness of vertebra within each group was not statistically different and ranged from 2583 N/mm in the control group to 2989 N/mm in the treatment group (*p* = 0.19) (Table 2).

**Table 2:** Results relative to bone mineral density, volume of the vertebral body, and mechanical testing (*N* = 7 per group)

	Control	Sham	Treatment	*p*
Bone mineral density (mg/cm^3^)	62.5 (48.6–77.6)	57.6 (43.1–77.9)	56.5 (44.9–70.8)	0.55
Volume (mL)	29.5 (18.9–40.3)	31.8 (21.1–38.8)	34.4 (28.4–44.5)	0.39
Stiffness before intervention (N/mm)	2583 (2071–3201)	2931 (2202–3492)	2989 (2591–3634)	0.19
Stiffness after intervention (N/mm)	2653 (2203–3185)	2815 (2221–3199)	3185 (2414–4067)	0.11
Failure load (N)	2841 (2010–3473)	2186 (1502–2878)	4118 (3373–5177)	< 0.001*
Displacement at failure (mm)	0.99 (0.75–1.30)	0.80 (0.58–1.08)	1.11 (0.95–1.26)	0.007**
Stiffness during load to failure test from 500 to 1500 N (N/mm)	3302 (2391–4212)	3403 (1747–4300)	4078 (3222–4814)	0.11

Mean and range are stated for all results

*p < 0.05 for: treatment vs. control, treatment vs. sham

**p < 0.05 for: treatment vs. sham

**Fig. 3:**
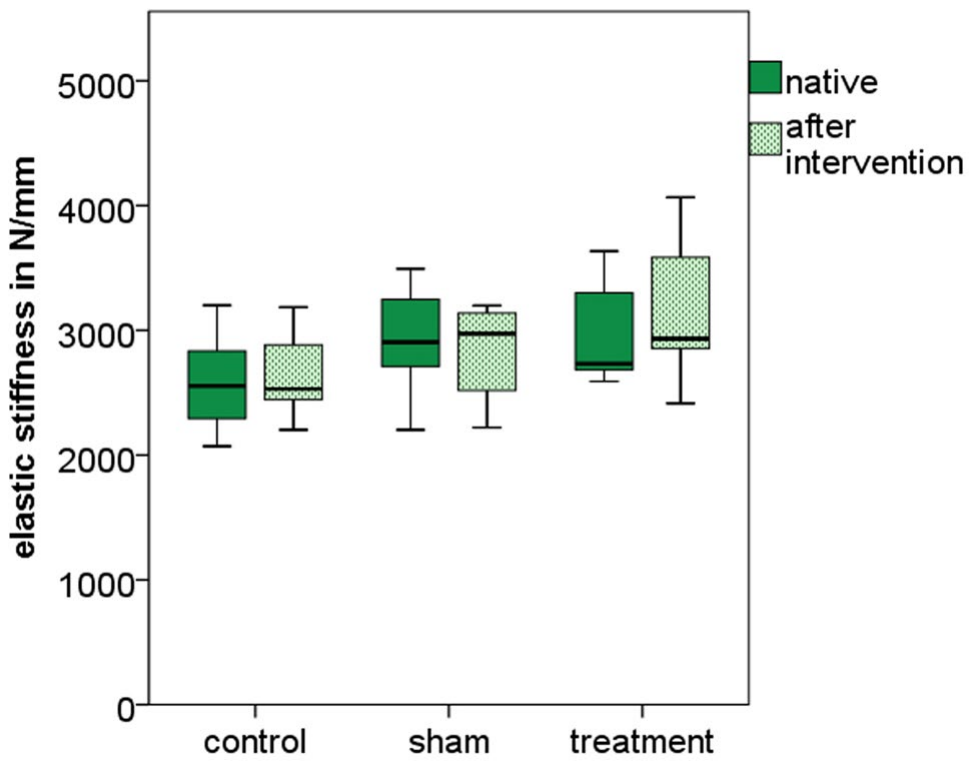
Boxplot of the pre and post interventional stiffness for the three test groups showing the median and quartiles

Total volume injected in the treatment group was 6.8 mL (5.7–8.9 mL) and the implant material was distributed in the anterior half of the vertebral body (Fig. 2). The elastic stiffness was not statistically different between the groups: control was 2653 N/mm (2203–3185 N/mm), sham was 2815 N/mm (2221–3199 N/mm), and treatment was 3185 N/mm (2414–4067 N/mm) (*p* = 0.11). There was no significant difference in stiffness before and after treatment in any group (paired t-test, *p* = 0.31) (Fig. 3).

**Fig. 4:**
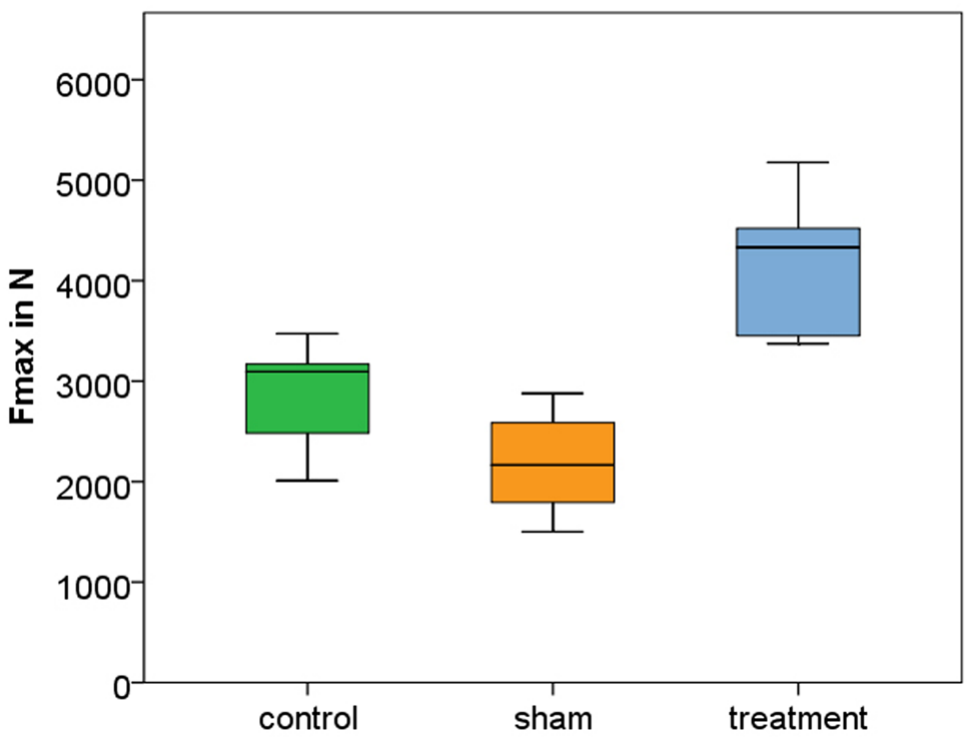
Boxplot of maximum failure load of the three test groups showing the median and quartiles

The failure load of the treatment group was statistically higher than either in the control or the sham group: 4118 N (3373–5177 N) in the treatment group compared to 2841 N (2010–3473 N) in the control group and 2186 N (1502–2878 N) in the sham group (Fig. 4). There was a significantly higher mean failure load in the treatment group in comparison to the control group (*p* = 0.003; [CI 451; 2103]) and to the sham group (*p* < 0.001; [CI 1105; 2758]) (Table 2).

The mean displacement at failure was higher for the treatment group (1.11 mm, range 0.95–1.26) compared to the control group (0.99 mm, range 0.75–1.30) and the sham group (0.80 mm, range 0.58–1.08) (Table 2). There was a significantly higher mean displacement at failure in the treatment group in comparison to the sham group (*p* = 0.005; [CI 0.09; 0.53]), but not between sham and control group (Table 2).

The mean stiffness during loading to failure from 500 to 1500 N was highest for the treatment group (4078 N/mm, range 3222–4814), but it was not significantly different than either the control group (3302 N/mm, range 2391–4212) or the sham group (3403 N/mm, range 1747–4300) (*p* = 0.11) (Table 2).

## Discussion

A therapeutic intervention that allows localized and immediate protection of osteoporotic bone is attractive for use in the osteoporotic spine where specific vertebra can be readily identified as being at a higher fracture risk either as a result of being adjacent to PMMA treatment of a painful index fracture or as a result of its position adjacent to long instrumentation used to correct a deformity. This study evaluated one such approach, a local osteo-enhancement procedure with a triphasic calcium-based implant material. In this study, the impact of the procedure on the immediate biomechanical properties of intact, i.e. non-fractured, cadaveric osteoporotic vertebra was evaluated. Following treatment, enhanced vertebra were significantly stronger (mean failure load of 4118 N) than either the control or sham groups (2841 N and 2186 N respectively). In contrast to the effect on failure load, treatment of the vertebra had no significant effect on stiffness either before or after intervention (no statistical differences between control, sham or treated vertebra). Thus, the treatment increased failure load without significantly increasing stiffness which reduces the risk of the treatment introducing a stress riser or stress shielding. These differences in biomechanical properties between groups was a result of the interventions used and not due to differences in donor age or bone mineral density of the vertebra since there were no significant differences in these parameters between groups (Table 2).

The mean failure load values reported here using intact osteoporotic vertebra are similar to values reported by others. For example, using fractured cadaveric vertebra Belkoff et al. studied failure loads of lumbar vertebral bodies (L1–L5) after vertebroplasty with three different polymethylmethacrylate cements and reported failure loads from 3584 to 6677 N [12]. In a separate study, the same group compared polymethylmethacrylate cement and hydroxyapatite cement for vertebroplasty of fractured human vertebra and observed failure loads from 2476 to 4146 N in T8–T10 and failure loads from 2450 to 4208 N in L2–L4 [18]. Furtado et al. studied the biomechanical properties of PMMA repaired fractured vertebra and intact osteoporotic vertebra prophylactically treated with PMMA [13]. They demonstrated no significant difference in failure load when PMMA vertebroplasty was used to treat osteoporotic vertebral compression fractures or when used prophylactically to treat intact vertebra (failure load of 2630 N for treated fractured vertebra and 2230 N for prophylactic treatment) [13]. This study demonstrated the immediate strengthening effect of the treatment procedure on osteoporotic vertebral bodies. In addition, use of the resorbable triphasic calcium-based implant material offers several advantages over PMMA for use in spine. As noted above, the material is less likely to introduce stress risers or stress shielding. As the material is resorbed and replaced with bone it provides a biological solution to the problem of bone loss due to osteoporosis that PMMA cannot. The treatment procedure also does not limit future interventions on the treated vertebra since it can be easily drilled or revised if needed.

The potential benefits of this approach in the spine must outweigh the potential risks of the elective interventional procedure. The published symptomatic complication rate of osteoporotic vertebral compression fracture augmentation with PMMA is 2–4% [19, 20]. These complications include infection, neural injury, and extraosseous cement leakage. Cement leakage is the most common adverse event, and although most leakages are asymptomatic, the most severe complications are cord compression and pulmonary embolism. The surgical technique used in the current study is designed to minimize implant leakage by preparing the vertebral body prior to injection with saline lavage. This technique has been shown to significantly reduce leakage frequency while improving cement distribution within the vertebral body [15]. It is hypothesized that saline lavage removes marrow, fat, and soft tissue elements from the vertebral body, leading to a measurable decrease in pulmonary fat emboli during cement injection into a vertebral body [21].

Recent publications describing the use of injectable calcium sulfate/calcium phosphate materials within fractured and intact vertebral bodies demonstrated no systemic complications due to implant leakage while achieving fracture reduction and maintenance of vertebral body height [22–24].

A limitation of the present study is the small number of replicates for each treatment group. While limited numbers of fresh frozen cadaver specimens were utilized for testing, care was taken to minimize differences in the known clinically relevant confounding variables (BMD, donor age, and volume). It was assumed that if a limited number of test samples did not show a statistical difference, the clinical impact of any difference would likely to be negligible and, therefore, an a priori sample size calculation was not conducted [25]. Another limitation of this study is that a cadaveric study can only investigate the immediate protection offered by the treatment to osteoporotic vertebra. In vivo, the material is resorbed and replaced with new bone and the effect of these changes on the biomechanical properties of the treated vertebra over time are outside the scope of this study. Future clinical studies will be needed to evaluate the longer term effects of the procedure.

In conclusion, the present pilot biomechanical study demonstrated that augmentation of intact osteoporotic vertebra using a triphasic calcium sulfate/calcium phosphate implant material significantly increased the failure load and displacement at failure compared to either native vertebra or vertebra that had undergone a sham procedure. Thus, local osteo-enhancement of osteoporotic vertebra with this implant material may offer meaningful benefits to patients by providing immediate protection of vertebra at risk of fracture. This cadaver study describes the first use of the local osteo-enhancement procedure with a triphasic calcium sulfate/calcium phosphate implant material in the spine.
